# Tailoring Microstructure and Mechanical Properties of the Al-7Si-0.35Mg-0.35Fe Alloy by Cr Addition: A Study on Fe-Rich Phase Modification

**DOI:** 10.3390/ma19030593

**Published:** 2026-02-03

**Authors:** Chiteng Le, Wenjun Liu, Tiancai Yin, Shuai Zhao, Cong Gao, Mingbo Yang, Tiehu Li, Bin Jiang

**Affiliations:** 1 College of Materials Science and Engineering, Chongqing University of Technology, Chongqing 400054, China; lechiteng@stu.cqut.edu.cn (C.L.); yintc@stu.cqut.edu.cn (T.Y.);; 2 Changan Auto Global R&D Center, Chongqing Changan Automobile Co., Ltd., Chongqing 401133, China; gaocong@changan.com.cn; 3 College of Materials Science and Engineering, Chongqing University, No. 174, Chongqing 400044, China

**Keywords:** Al-7Si alloy, Cr, Fe-rich phase, fracture morphology

## Abstract

Fe-rich phases are unavoidable intermetallic compounds in aluminum alloys, particularly in recycled aluminum. Their needle-like morphology not only impairs the mechanical performance of the alloy by disrupting the continuity of the matrix but also significantly reduces the allowable addition of recycled aluminum materials. Based on this, this study focuses on the Al-7Si-0.35Mg-0.35Fe alloy with a high Fe content. The Cr was introduced to modify the characteristics of the Fe-rich phase, and the microstructural evolution and mechanical properties of the aluminum alloy with different Cr content (0–0.25 wt.%) were investigated. Experimental results show that the secondary dendrite arm spacing of the alloy is significantly refined after Cr addition. Meanwhile, the Fe-rich phase gradually transitions from β-Al_5_FeSi with needle-like morphology to α-Al_15_(Fe,Cr)_3_Si_2_ with short rod-like or blocky morphology as the Cr content increases. Notably, the Fe-rich phase in the 0.20Cr alloy exhibits an approximately 65% increase in sphericity and an 84% reduction in equivalent diameter compared to those in the 0Cr alloy. The morphological blunting and dispersed distribution of Fe-rich phases lead to a broad effective Cr addition range of 0.05–0.20 wt% in the alloy. Among them, the 0.20Cr alloy exhibited the best comprehensive mechanical properties, with its ultimate tensile strength and elongation approximately 19% and 107% higher than those of the 0Cr alloy, respectively. Furthermore, the fracture morphology and the relationship between the Fe-rich phase and microcracks in Al-7Si-0.35Mg-0.35Fe alloys with different Cr contents were also analyzed.

## 1. Introduction

The Al-7Si family of aluminum alloys, such as A356 and A319, is widely utilized in the production of structurally demanding components for automotive and aerospace applications. This prevalence is attributed to their excellent castability, favorable corrosion behavior, relatively high specific strength, and effective recyclability [1,2,3]. As component lightweighting advances relentlessly, the widespread adoption of recycled aluminum has become standard industry practice, driven by cost control and resource sustainability. However, impurity elements like Fe inevitably enter recycled aluminum alloys. Among these, Fe exhibits extremely low solid solubility in aluminum: at the eutectic temperature of 655 °C, its maximum solid solubility is merely about 0.05 wt%. Furthermore, during the solidification, Fe readily forms various hard and brittle intermetallic compounds with aluminum, silicon, and elements like Mn, Cr, and Mg, which are collectively known as Fe-rich phases. The most prevalent Fe-rich phases include the needle-like β-Fe phase and the blocky or Chinese script-like α-Fe phase. These Fe-rich phases, particularly the needle-like β-Fe, severely damage the continuity of the matrix and reduce the mechanical properties of the alloy [4,5,6,7,8,9,10,11]. They also dramatically deteriorate the alloy’s casting performance and impair its subsequent machinability [12,13]. It should be noted that the detrimental effects and tolerance limit of Fe impurities in Al-Si alloys are strongly dependent on the Si content and its availability in the matrix. Recent studies have demonstrated that alloys with higher effective Si content exhibit improved tolerance to elevated Fe levels, owing to altered solidification behavior and Fe-rich intermetallic evolution [14,15,16]. In contrast, hypoeutectic Al-7Si systems remain particularly sensitive to Fe-induced β-Al_5_FeSi formation, and conclusions drawn from high-Si systems cannot be directly transferred to Al-7Si-based alloys. Therefore, composition-specific investigations are required to clarify Fe-rich phase behavior in Al-7Si alloys with elevated Fe contents. Thus, with Fe impurities inevitably present, the critical technical challenge lies in effectively controlling and modifying the morphology, size, and distribution of Fe-rich phases, thereby minimizing their detrimental effects to enhance the comprehensive performance of Al-7Si alloys and promote the broader utilization of recycled aluminum [17,18].

Scholars have conducted extensive research on modifying the Fe-rich phases, focusing primarily on two approaches, namely solidification process control and alloying regulation. Solidification process control mainly involves melt treatment techniques, like rapid cooling [19], electromagnetic stirring [20], and ultrasonic treatment [21], to influence the precipitation behavior of the Fe-rich phase. This refines both the aluminum matrix and the Fe-rich phases, thus partially suppressing their harmful effects. Alloying regulation, however, can fundamentally alter the crystallization path and growth kinetics of the Fe-rich phase by introducing “neutralizing” elements such as Mn [8,22,23,24,25], Cr [25,26,27,28], Mo [29,30,31,32], V [33,34], Be [35], Sc [36,37,38], and RE [39,40,41]. It has become the industry’s most prevalent method to drive the transformation of the harmful needle-like β-Fe phase into the less detrimental blocky or Chinese script-like α-Fe phase. Among these, transition metals such as Mo and V exert their influence primarily by forming Al(Fe,Mo)Si [29] and Al_12_(Fe,V)_3_Si [34] phases. This behavior alters the nucleation and growth of Fe-rich phases to some extent, transforming their morphology from needle-like into plate-like or blunting polyhedral structures. Rare earth (RE) elements, such as Ce [40], La, and Y [41], create rare earth-rich atomic layers through their segregation at the grain and phase boundaries. These layers inhibit the oriented growth of the β-Al_5_FeSi phase, promoting its morphological blunting. Simultaneously, they refine α-Al dendrite structures, significantly enhancing the alloy’s mechanical properties. However, the high cost, challenging process control, and narrow compositional window limit the practical application of both transition elements and RE elements in production. Accordingly, Mn and Cr, which balance cost-effectiveness with process stability, have emerged as the preferred alternatives for modifying the needle-like Fe-rich phase in aluminum alloys.

In Al-Si series alloys, Mn [8] addition can increase the formation of α-type phase (e.g., α-Al_15_(Fe,Mn)_3_Si_2_) in microstructure and facilitate their precipitation as fine and dispersed Chinese script-like or star-like morphologies. This concurrently enhances the strength and plasticity of the aluminum alloy. However, the Mn content must be strictly controlled. Insufficient Mn leaves a significant amount of untransformed needle-like β-Fe phase in the microstructure, while excess Mn leads to coarsened α-Fe phases, which markedly reduce the service life of molds. Therefore, in die-casting alloys, the Mn content is usually controlled at 0.1 wt.% or lower. Compared with Mn, Cr possesses higher electronegativity and bonding characteristics, resulting in stronger chemical affinity with Al and Si atoms. Furthermore, its 3D electron orbitals favor the formation of metallic bonds with a higher proportion of stronger and more stable covalent components. This enhances the nucleation thermodynamic stability of Cr-containing α-Fe phases, effectively suppressing the precipitation of metastable needle-like β-Fe phases during solidification, and Cr is consequently regarded as a potential substitute for Mn. Balasubramani et al. [25] found that the combined addition of Mn (0.25, 0.5, and 0.8 wt.%) and Cr (0.15–0.20 wt.%) transformed all needle-like Fe-rich phases into blunting α-Fe phases, thereby effectively improving the mechanical properties of the alloy. Gustafsson et al. [27] further corroborated that Cr acts as an effective “neutralizer” for Fe-rich phases and facilitates α-Fe nucleation. However, most of the studies mainly focus on aluminum alloys with iron content not exceeding 0.15 wt.%, while studies on Al-7Si systems with Fe contents exceeding 0.15 wt.% remain limited. Moreover, there is a current lack of systematic investigation into the morphology and distribution characteristics of Fe-rich phases under varying Cr contents, as well as their underlying influence mechanisms on crack propagation behavior.

In summary, Cr demonstrates a favorable effect on Fe-rich phases in aluminum alloys with low iron content. However, to further elevate the permissible iron content limits, it is essential to investigate the influence of Cr microalloying on the microstructural evolution and mechanical properties of aluminum alloys with high iron content. This necessitates a focused analysis of the controlling mechanisms of Fe-rich phases, alongside the nucleation and propagation of deformation cracks under these mechanisms. Such research will lay a foundation for expanding the application of recycled aluminum.

## 2. Experimental Materials and Methods

### 2.1. Materials

**Figure 1:**
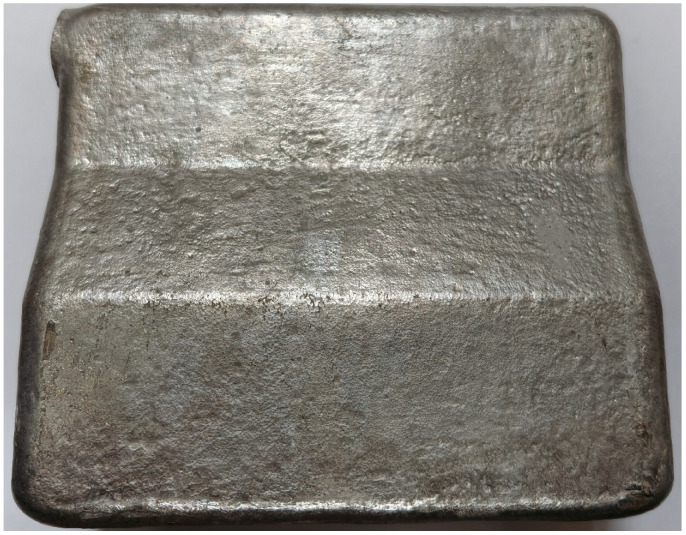
Macroscopic structure diagram of the casting body.

The test alloy was prepared from industrial pure aluminum with 99.70% purity and the master alloys of Al-10Si, Al-10Mg, Al-10Fe, Al-10Cr, Al-10Ti, and Al-10Sr, which were purchased from Northeast Nonferrous Metals Company (Shenyang, China). All alloy compositions were expressed by mass fraction. The raw materials were used after the oxide film was removed and dried. Firstly, pure aluminum was placed in a resistance furnace and heated to 700 °C to melt. Then, Al-10Si was added, and the temperature was reduced to 600 °C to avoid overheating of the melt and burning loss of alloying elements caused by the heat release of Si melting. When the base material had melted, the melt temperature was elevated to 700 °C. Thirdly, the master alloys of Al-10Fe, Al-10Cr, and Al-10Ti were introduced sequentially into the melt, with the entire process conducted under a protective atmosphere of high-purity argon to prevent oxidation and contamination. Accordingly, the melt temperature was raised to 720 °C and held for 15 min. After that, the melt was raised, stirred, and refined with hexachloroethane for 1–2 min at 730 °C. After refining and slag removal, the Al-10Mg and Al-10Sr were added at 720 °C. The purpose was to utilize the relatively low temperature and clean melt environment to reduce the oxidation and volatilization losses of Mg and Sr. Subsequently, the completely melted alloy was held at this temperature for 20 min, and then the slag was removed. Finally, the prepared melt is poured into a mold preheated to 250 °C, and the formed ingot (127 × 49 × 110 mm) is shown in Figure 1. The final chemical composition of the alloy was analyzed using inductively coupled plasma optical emission spectrometry (ICP), with results averaged over three independent measurements to ensure data reliability. The specific composition is shown in Table 1. The actual measured Cr content fell below the nominal composition, suggesting partial loss of Cr during melting, likely due to its relatively high chemical reactivity and associated volatilization or oxidation.

### 2.2. Analysis Method

The metallographic microstructure of the alloys was examined using a Leica DMI5000M optical microscope (Leica Microsystems, Wetzlar, Germany). To reveal the microstructure, specimens were subjected to etching in Keller’s reagent (composed of 1 mL HF acid, 1.5 mL HCl acid, 2.5 mL HNO_3_, and 95 mL H_2_O) for a duration of 10 to 15 s. The morphology and distribution of the Fe-rich phase, as well as the tensile fracture surfaces, were further characterized using a scanning electron microscope (SEM, Thermo Fisher Apreo 2S; Thermo Fisher Scientific, Waltham, MA, USA) equipped with energy-dispersive X-ray spectroscopy (EDS). X-ray diffraction (XRD) with Cu Kα radiation (Panalytical Empyrean Series 2 diffractometer; Panalytical, Almelo, The Netherlands) was employed for phase analysis, with a 2θ scanning range from 20° to 90° to identify the crystalline constituents in the alloy. Morphological observations and elemental mapping were carried out via transmission electron microscopy (TEM) on an FEI Tecnai G2 F20 instrument (FEI, Hillsboro, OR, USA), integrated with a Bruker energy-dispersive X-ray spectroscopy (EDS, Bruker Corporation, Billerica, MA, USA) system for detailed microstructural and compositional analysis.

Room-temperature tensile tests were carried out using a WDW300 electronic universal testing machine (Jinan Hanshen Precision Instrument Co., Ltd., Jinan, China). The tensile samples were machined with a gauge length of 25 mm and a uniform parallel section diameter of 5 mm. All tensile tests were conducted at a constant displacement rate of 1 mm/min using a universal testing machine. For each alloy variant, three nominally identical specimens were tested, and the reported mechanical properties were calculated as the mean value of the valid results to ensure statistical reliability.

## 3. Results and Discussion

**Figure 2:**
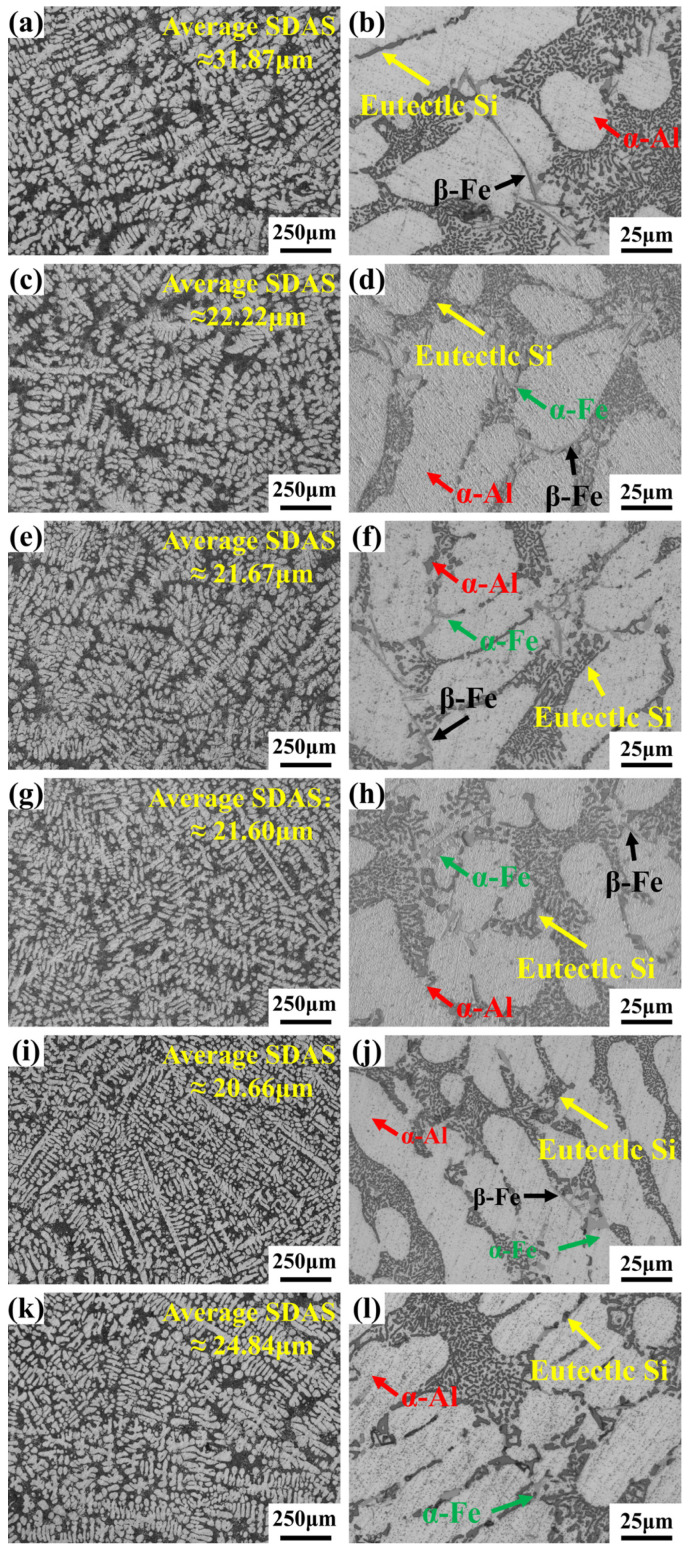
Optical microstructures of Al-7Si-0.35Mg-0.35Fe alloys with different Cr contents: (**a**,**b**) 0Cr; (**c**,**d**) 0.05Cr; (**e**,**f**) 0.10Cr; (**g**,**h**) 0.15Cr; (**i**,**j**) 0.20Cr; (**k**,**l**) 0.25Cr.

Figure 2 shows the optical microstructures of as-cast Al-7Si-0.35Mg-0.35Fe alloys with varying Cr contents (0–0.25 wt.%). The microstructure primarily consists of primary α-Al dendrites (white), eutectic silicon (light gray), and Fe-rich phase (dark gray). With increasing Cr content, the secondary dendrite arm spacing (SDAS) initially decreased and then increased. The SDAS reached its minimum value of 20.66 μm in the 0.20Cr alloy, approximately 35% lower than that of the 0Cr alloy. However, further increasing the Cr content to 0.25 wt.% paradoxically coarsened the microstructure relative to the 0.20Cr alloy, increasing SDAS by 20.23%, yet still maintaining a 22.06% reduction compared to the 0Cr alloy. Meanwhile, Cr addition profoundly influenced the morphology and distribution of the Fe-rich phase. In the 0Cr alloy (Figure 2a,b), numerous needle-like β-Fe phases were present. As Cr content increased, the Fe-rich phase progressively transformed from the β-Fe phase [42] to the α-Fe phase [43]. Within the 0.05–0.20 wt.% Cr range (Figure 2c–i), the quantity of β-Fe phase continuously decreased, steadily replaced by finer and more regularly shaped α-Fe phase. Further increasing the Cr content to 0.25 wt.% (Figure 2k,l) induced some coarsening of the Fe-rich phase. Thus, for high Fe content aluminum alloys, Cr addition effectively improves the microstructure of the alloy. It refines the aluminum matrix with a smaller SDAS, while simultaneously promoting the transformation of detrimental β-Fe phase into the more favorable α-Fe phase. Nevertheless, excessive Cr (0.25 wt.%) causes both the SDAS and Fe-rich phase to coarsen.

**Figure 3:**
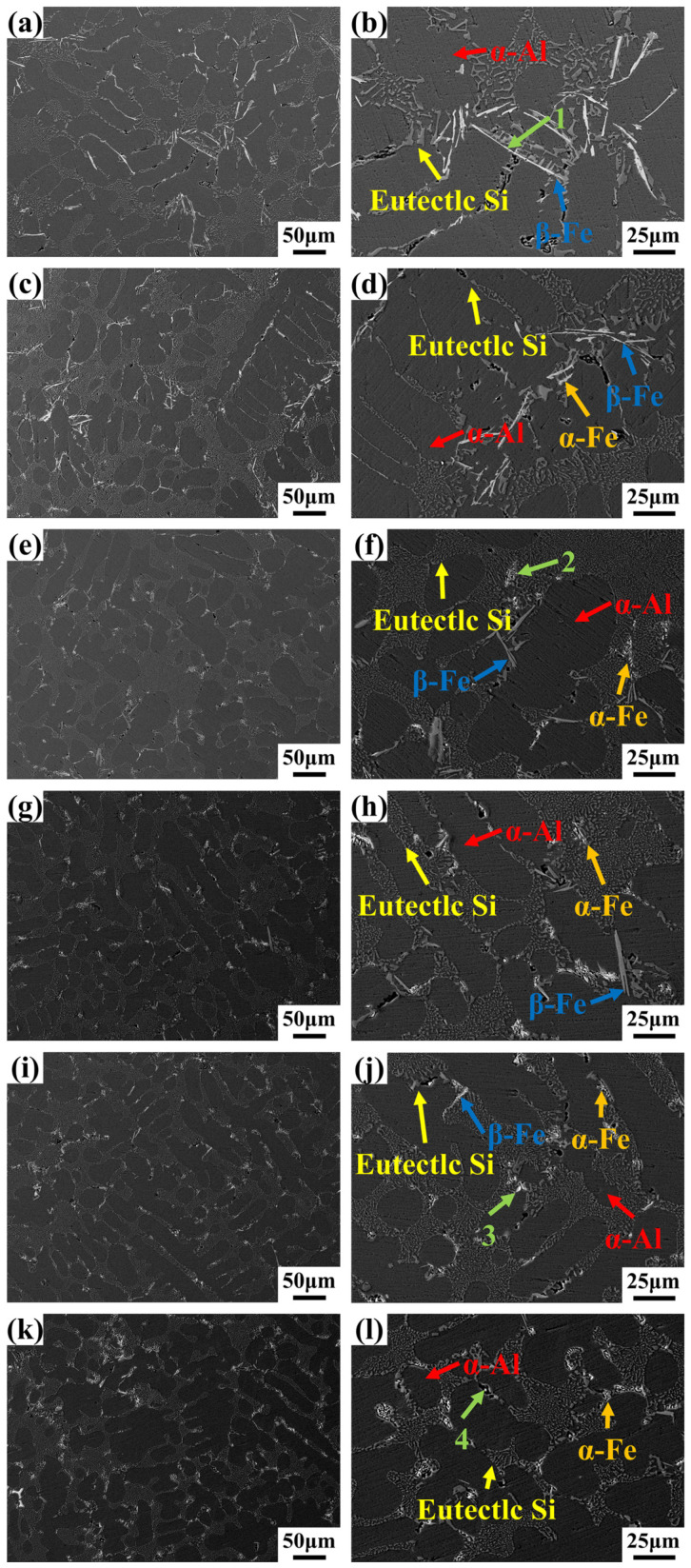
SEM images of Al-7Si-0.35Mg-0.35Fe alloys with different Cr contents: (**a**,**b**) 0Cr; (**c**,**d**) 0.05Cr; (**e**,**f**) 0.10Cr; (**g**,**h**) 0.15Cr; (**i**,**j**) 0.20Cr; (**k**,**l**) 0.25Cr.

**Figure 4:**
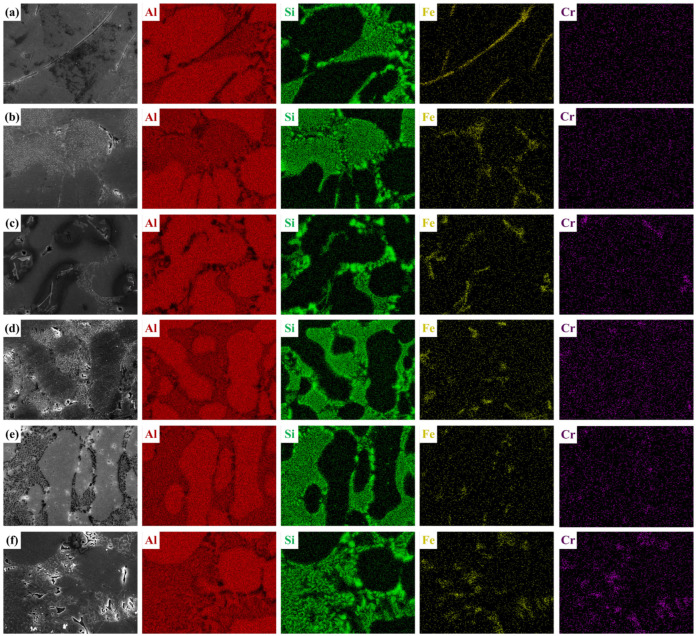
SEM images and corresponding EDS elemental mappings of representative Fe-rich phases: (**a**) 0Cr; (**b**) 0.05Cr; (**c**) 0.10Cr; (**d**) 0.15Cr; (**e**) 0.20Cr; (**f**) 0.25Cr.

The SEM morphology of as-cast Al-7Si-0.35Mg-0.35Fe alloys with different Cr contents is presented in Figure 3. Chemical compositions of Fe-rich regions with characteristic morphologies were determined using EDS (Thermo Fisher Scientific, Waltham, MA, USA). The designated points 1–4 mark the positions where EDS measurements were conducted (refer to Table 2). As Cr content increases, the morphology of the Fe-rich phases undergoes a typical evolution, transforming from needle-like to rod-like and finally to blocky. In 0Cr alloys, the Fe-rich phases mainly appear near eutectic Si with needle-like morphology. The EDS atomic ratio of Al:Si:Fe approximates 5:1:1, aligning with the β-Al_5_FeSi phase [44]. With 0.05 wt.% Cr addition, some needle-like β-Fe phase was replaced by a short rod-like Fe-rich phase. With Cr increased to 0.10 wt.%, the needle-like β-Fe phase was significantly reduced, while more short rod-like Fe-rich phases and a minor quantity of blocky Fe-rich phases appeared in the microstructure. As Cr further increased to 0.15–0.25 wt.%, the blocky Fe-rich phases emerged as the dominant morphology, with only trace amounts of extremely needle-like β-Fe phases remaining. To elucidate the nature of the newly formed short rod-like Fe-rich phases (Point 2) and blocky Fe-rich phases (Points 3 and 4), EDS point analyses were conducted on these representative regions. The short rod-like Fe-rich phase at Point 2 exhibits an atomic composition of Al 87.69 at.%, Si 5.60 at.%, Fe 4.98 at.%, and Cr 1.73 at.%, indicating a reduced Si content and the incorporation of Cr into the Fe-rich phase lattice. Similarly, the blocky Fe-rich phases at Points 3 and 4 show comparable compositional characteristics, namely Fe enrichment, relatively low Si content, and the presence of Cr. These compositional features confirm that both the short rod-like and blocky Fe-rich phases correspond to α-Al(Fe,Cr)Si phases, demonstrating a Cr-induced structural transition from β-Fe phases to α-Fe phases [45]. To further comprehensively evaluate the elemental distribution characteristics of Fe-rich phases with different morphologies, EDS elemental mapping was performed on representative needle-like, short rod-like, and blocky phases, as shown in Figure 4. The needle-like Fe-rich phases exhibited elemental distributions dominated by Al, Si, and Fe, with no detectable Cr enrichment, indicating that these phases correspond to the β-Fe phases. In contrast, both the short rod-like and blocky Fe-rich phases showed the obvious Cr enrichment feature, with the element distribution mainly composed of Al, Si, Fe, and Cr, indicating that Cr has participated in the formation of the Fe-rich phases. Based on the aforementioned point scanning results, it could be confirmed that both the short rod-shaped and block-shaped Fe-rich phases were α-Fe phases. This result further confirmed that the morphological transition from needle-like to short rod-like and blocky phases was accompanied by a compositional evolution from Cr-free β-Fe phases to Cr-containing α-Fe phases. This progression clearly indicates that the Cr addition promoted the precipitation of the α-Fe phase, while inhibiting the precipitation and growth of the β-Fe phase.

**Figure 5:**
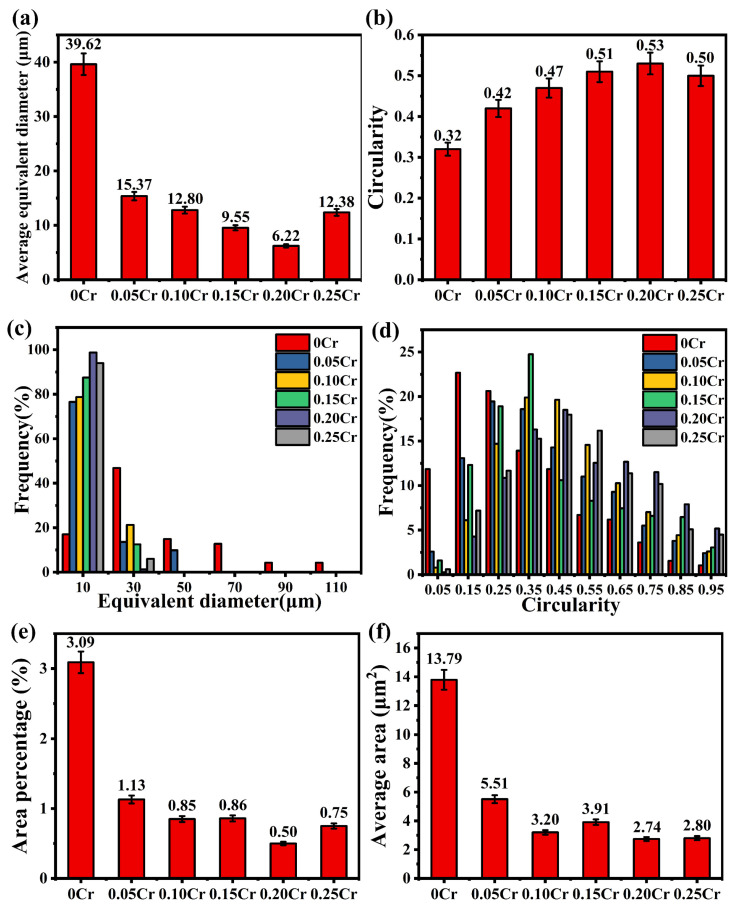
Statistical analysis of Fe-rich phases in Al-7Si-0.35Mg-0.35Fe alloys with different Cr contents: (**a**) average equivalent diameter of Fe-rich phase; (**b**) average sphericity of Fe-rich phase; (**c**) equivalent diameter distribution of Fe-rich phase; (**d**) sphericity distribution of Fe-rich phase; (**e**) area percentage of individual Fe-rich phases; (**f**) average area of individual Fe-rich phases.

In order to gain deeper insight into the influence of Cr addition on the morphology of Fe-bearing intermetallic phases within high-Fe Al-7Si-0.35Mg-0.35Fe alloys, a quantitative microstructural analysis was conducted, focusing on parameters such as equivalent diameter and circularity [46]. The analysis was performed on the Fe-rich phase observed in SEM images; the results are shown in Figure 5. As Cr content increases, the equivalent diameter of Fe-rich phases shows an initial decrease followed by a slight increase (Figure 5a). The minimum value is reached in the 0.20Cr alloy at 6.22 μm, representing a reduction of approximately 84% compared to the 0Cr alloy. It can be known from Figure 5c that it is mainly distributed around 10 μm. When the Cr content is further increased to 0.25 wt.%, the equivalent diameter rises slightly but remains 68.75% lower than that of the 0Cr alloy. This indicates that its inhibitory ability on the growth and coarsening of the Fe-rich phase continues to enhance. Furthermore, the closer the circularity is to 1, the larger the radius of curvature at the edge of the Fe-rich phase, which can significantly reduce the interfacial stress concentration factor (Kt) from a mechanical perspective. With the increase in Cr content, the average sphericity of the Fe-rich phases shows a trend of rising and then slightly decreasing, reaching the maximum value of 0.53 at 0.20Cr, which is approximately 66% higher than that at 0Cr. The circularity distribution plot (Figure 5d) shows that the number of Fe-rich phases with circularity > 0.5 increases noticeably within the 0.15–0.20Cr range. When the Cr content is further raised to 0.25 wt.%, the average circularity slightly drops to 0.50 (about 5.7% lower than that of the 0.20Cr alloy). Therefore, the addition of Cr can significantly reduce the size of the Fe-rich phase and enhance its roundness, achieving dual optimization of “size refinement and morphological rounding,” thereby mitigating the detrimental influence of the Fe-rich phase. Based on the morphological evolution, the area percentage and average area of individual Fe-rich phases were calculated, as shown in Figure 5e,f: The area fraction and average area of individual Fe-rich phases in 0Cr alloy were 13.79 μm^2^ and 3.09%, respectively. As the Cr content increased from 0.05 to 0.25 wt.%, the area fraction and average area of Fe-rich phases showed a trend of first decreasing and then increasing. Among them, the area fraction and average area of individual Fe-rich phases in the 0.20% Cr alloy were the smallest, being 2.74 μm^2^ and 0.50%, which were 80% and 84% larger than those of the individual Fe-rich phases in the 0Cr alloy. Therefore, the addition of Cr not only promoted the formation of the α-Fe phase but also effectively inhibited the directional growth of the β-Fe phase, thereby forming rounder, smaller, and more uniformly distributed Fe-rich phases.

**Figure 6:**
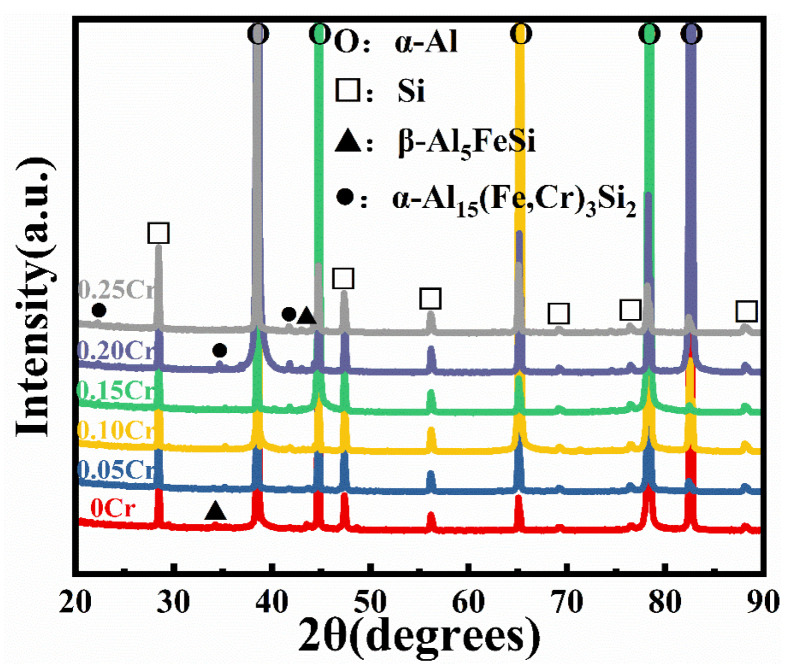
XRD patterns of Al-7Si-0.35Mg-0.35Fe alloys with different Cr contents.

Figure 6 shows the XRD spectra corresponding to alloys with varying Cr concentrations presented in the figure. In the absence of Cr, the dominant phases identified include the α-Al matrix, the eutectic Si, and the β-Al_5_FeSi intermetallic compound. However, after the addition of the Cr element, a new phase, α-Al_15_(Fe,Cr)_3_Si_2_, was introduced into the microstructure. With increasing Cr content, the diffraction peak intensity of the β-Al_5_FeSi phase decreased, while that of the newly formed α-Al_15_(Fe,Cr)_3_Si_2_ phase gradually increased. This phenomenon indicates that Cr addition leads to the substitution of Fe atoms by Cr atoms in the crystal lattice of the original β-Al_5_FeSi phase, or Cr atoms co-occupy certain lattice sites with Fe atoms [27]. Consequently, the formation kinetics of the β-Al_5_FeSi phases are notably restrained, thereby favoring the development of the α-Al_15_(Fe,Cr)_3_Si_2_ intermetallic.

**Figure 7:**
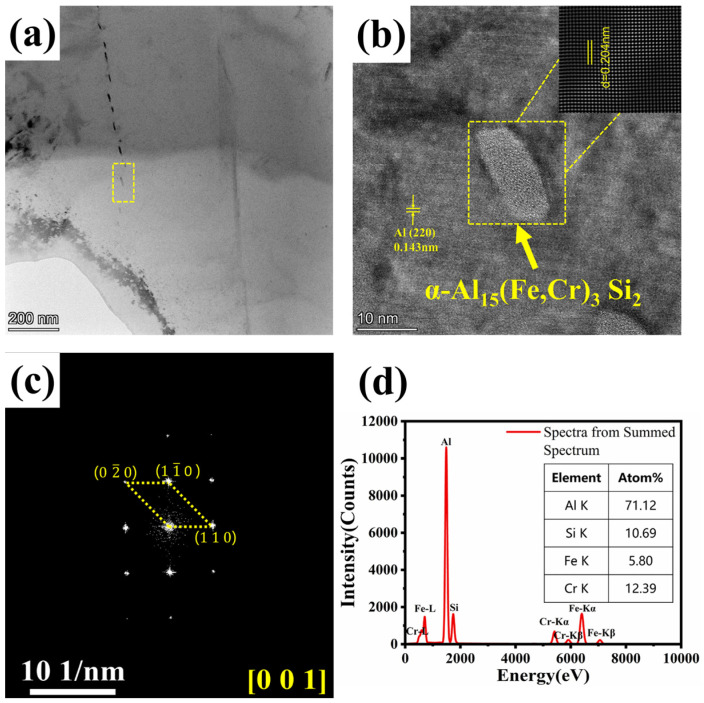
Identification of the α-Fe phase in Cr-containing alloys: (**a**) TEM bright-field image, (**b**) HRTEM image with marked interplanar spacings, (**c**) fast Fourier transform (FFT) pattern of the second phase, (**d**) EDS point analysis spectrum of the second phase in (**b**).

To clarify the bonding characteristics between the precipitates and the matrix, transmission electron microscopy (TEM) analysis was performed on the precipitates in the 0.20Cr alloy, and the results are shown in Figure 7. In the bright-field image (Figure 7a), short rod-like precipitates are clearly visible, with lengths of 14–19 nm and widths of 4–7 nm, dispersed mainly along the grain boundaries. Combined with the high-resolution image of a short rod-like precipitate (Figure 7b), the interplanar spacing was measured as 0.204 nm. Fast Fourier transform (FFT) was applied to the precipitate region (Figure 7c), yielding clear diffraction spots. Indexing confirmed that the diffraction pattern matched the [001] zone-axis pattern of the α-Al_15_(Fe,Cr)_3_Si_2_ phase. EDS point analysis (Figure 7d) further verified the composition of the precipitate, showing strong peaks of Al, Fe, Cr, and Si. EDS point analysis revealed that the atomic ratio of Al, (Fe,Cr), and Si was close to 15:3:2 (Al: 71.12 at.%, Si: 10.69 at.%, Fe: 5.80 at.%, Cr: 12.39 at.%). Integrating the EDS and FFT results, the short-rod precipitate was confirmed as α-Al_15_(Fe,Cr)_3_Si_2_. Based on crystallographic parameters, the lattice misfit between the (6 2 0) plane of α-Al_15_(Fe,Cr)_3_Si_2_ and the (2 2 0) plane of α-Al was calculated to be 42.66%, indicating that the interface is unlikely to form a coherent or semi-coherent structure but is predominantly incoherent. The high misfit results in a semi-coherent or incoherent interface structure, which hinders effective stress transfer across the boundary. Meanwhile, such interfaces also act as potent barriers to dislocation motion, leading to a significantly increased dislocation density in their vicinity. As a result, cracks tend to initiate and propagate preferentially at such interfaces. It is worth emphasizing that although the misfit between α-Al_15_(Fe,Cr)_3_Si_2_ and the matrix remains substantial, it actually shows improved interfacial compatibility compared with the β-Al_5_FeSi phase. According to interplanar spacing calculations, the lattice mismatch between β-Al_5_FeSi and the α-Al matrix is approximately 53%, significantly higher than the 42.66% for α-Al_15_(Fe,Cr)_3_Si_2_. Based on crystallographic parameters, the misfit of close-packed planes between α-Al_15_(Fe,Cr)_3_Si_2_ or β-Al_5_FeSi and α-Al was evaluated, revealing that when β-Al_5_FeSi transforms into α-Al_15_(Fe,Cr)_3_Si_2_, the misfit between the Fe-rich phase and α-Al is reduced by about 20%. It is well established that lattice mismatch tends to significantly increase dislocation density at the interface, impeding effective stress transfer. Therefore, compared with the α-Al_15_(Fe,Cr)_3_Si_2_/α-Al interface, cracks are more likely to nucleate at the β-Al_5_FeSi/α-Al interface.

**Figure 8:**
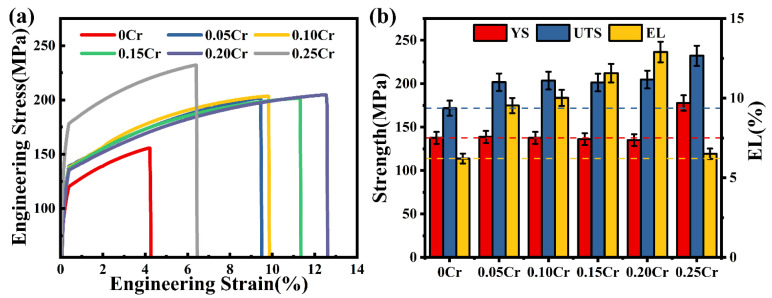
Mechanical properties of Al-7Si-0.35Mg-0.35Fe alloys: (**a**) engineering stress–strain curves, (**b**) variations in tensile properties.

Figure 8 presents the engineering stress–strain curves and corresponding tensile properties of Al-7Si-0.35Mg-0.35Fe alloys with different Cr contents. All Cr-containing alloys exhibited superior mechanical properties compared with the 0Cr alloys. As Cr content increased in the range of 0–0.20 wt.%, the alloy’s elongation (EL) significantly increased, while the ultimate tensile strength (UTS) and yield strength (YS) remained nearly unchanged. However, elevating the Cr content to 0.25 wt.% triggered a notable decline in elongation, accompanied by a marked increase in both UTS and YS. Among the studied alloys, the 0.20Cr alloy shows the optimal balance of strength and ductility, with YS, UTS, and EL values of 135.08 MPa, 204.79 MPa, and 12.90%, respectively. Compared to the 0Cr alloy, the 0.20Cr alloy achieves approximately a 19% increase in UTS and a 107% increase in EL. The notable enhancement in EL of these alloys with Cr content ranging from 0.05 to 0.20 wt.% is primarily attributed to the transformation of the β-Al_5_FeSi phase into the α-Al_15_(Fe,Cr)_3_Si_2_ phase. This transformation diminishes the likelihood of interfacial stress concentration and thereby suppresses the nucleation and propagation of interphase cracks during deformation. When the Cr content was further increased to 0.25 wt.%, the α-Al_15_(Fe,Cr)_3_Si_2_ phases coarsened, resulting in increased strength but a substantial loss of ductility. The corresponding YS, UTS, and EL of the 0.25Cr alloy were 177.86 MPa, 232.15 MPa, and 6.51%, respectively. Nevertheless, relative to the 0Cr alloy, the 0.25Cr alloy still exhibited increases of 29.17% in YS, 35.06% in UTS, and 4.83% in EL. Therefore, Cr additions in the range of 0.05–0.20 wt.% effectively enhanced the mechanical properties of the alloy. However, excessive addition (0.25 wt.%) led to the coarsening and segregation of Fe-rich phases, which embrittles the matrix. Thus, the alloy’s strength increased, while ductility was notably compromised.

**Figure 9:**
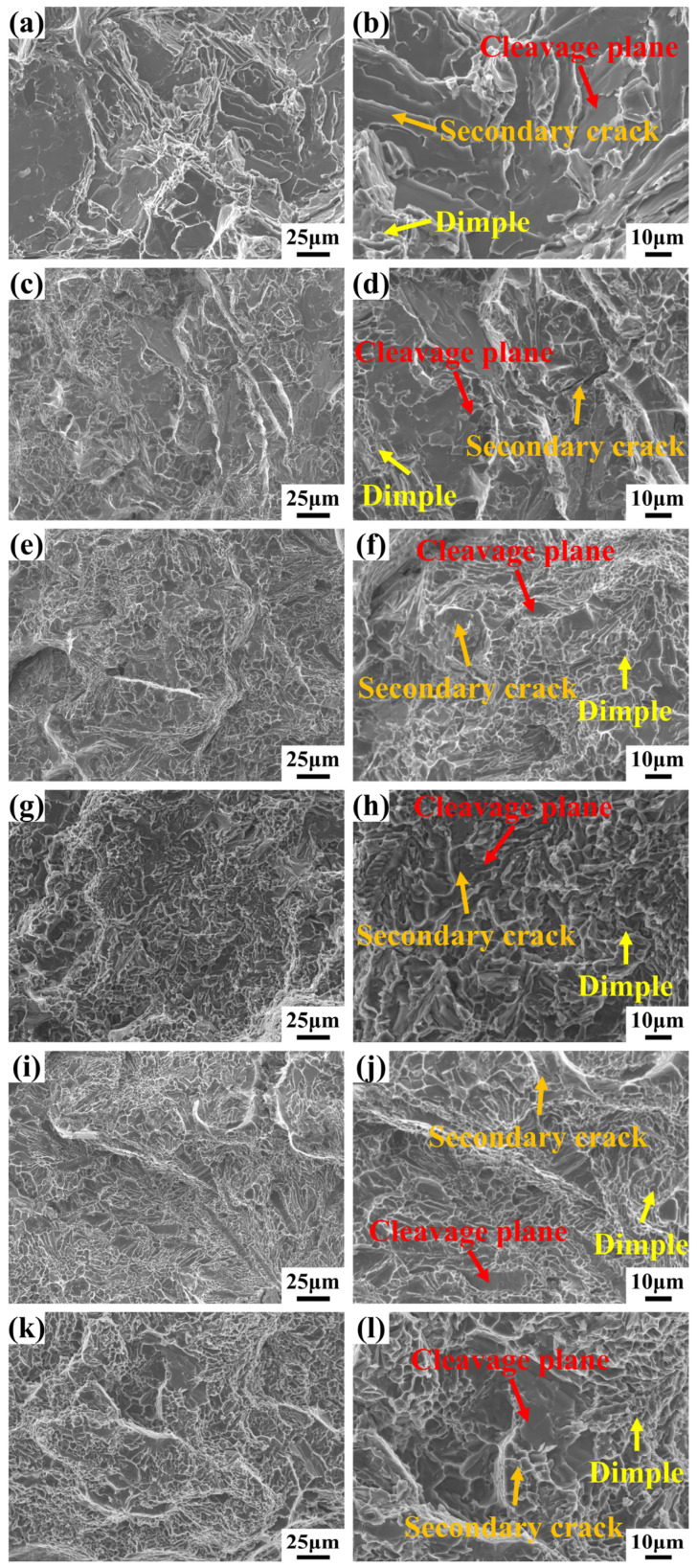
Fracture morphology of Al-7Si-0.35Mg-0.35Fe with varying Cr: (**a**,**b**) 0Cr; (**c**,**d**) 0.05Cr; (**e**,**f**) 0.10Cr; (**g**,**h**) 0.15Cr; (**i**,**j**) 0.20Cr; (**k**,**l**) 0.25Cr.

The fracture morphologies of Al-7Si-0.35Mg-0.35Fe alloys with different Cr contents were examined, and the results were presented in Figure 9. The fracture surface of the 0Cr alloy (Figure 9a) featured a large cleavage facet and microcracks, exhibiting brittle fracture characteristics. Following Cr addition, the fracture surface was mainly composed of small cleavage facets, microcracks, and dimples. Increasing Cr content significantly decreased both the number and size of cleavage facets, while simultaneously increasing the number of dimples and refining their size. Notably, at Cr contents of 0.10–0.20 wt.%, the fracture surface displayed more dimples and fewer cleavage facets, indicating a transition from brittle fracture to quasi-cleavage fracture, accompanied by a reduction in microcracks. The evolution that cleavage facets reduce and dimples proliferate perfectly aligns with the microstructure changes observed in Figure 3, where the morphology of Fe-rich phases transforms from needle-like to rod-like and blocky. However, when the Cr content increased to 0.25 wt.% (Figure 9k), the fracture surface features deep dimples in some areas and shallower cleavage facets and microcracks in other areas. This is attributed to the slight agglomeration and local coarsening of Fe-rich phases in the 0.25Cr alloy (reflected by the slight rebound in equivalent diameter and a slight decrease in circularity), which reintroduces localized stress-concentration sites in certain regions, promoting brittle crack propagation there. Therefore, the transformation of needle-like β-Al_5_FeSi to short rod-like or blocky α-Al_15_(Fe,Cr)_3_Si_2_ phase brought about by the addition of the Cr element significantly reduced the potential nucleation points of cracks, thereby enhancing the plastic deformation capacity of the alloy.

**Figure 10:**
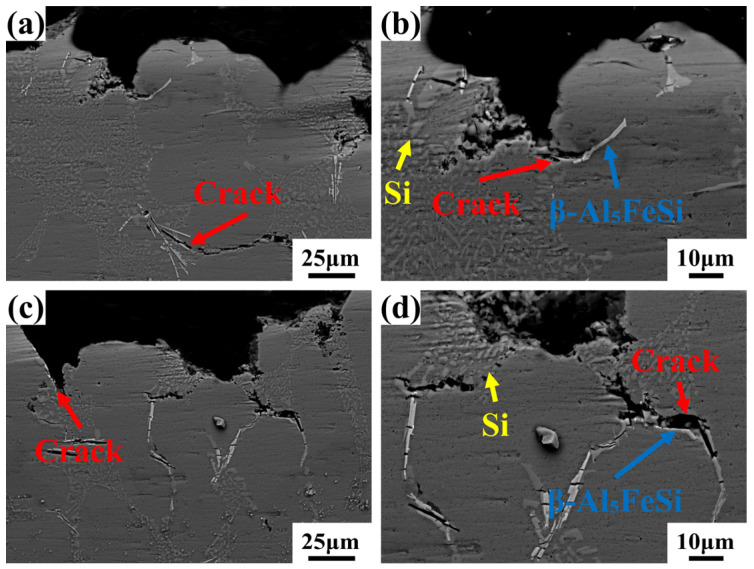

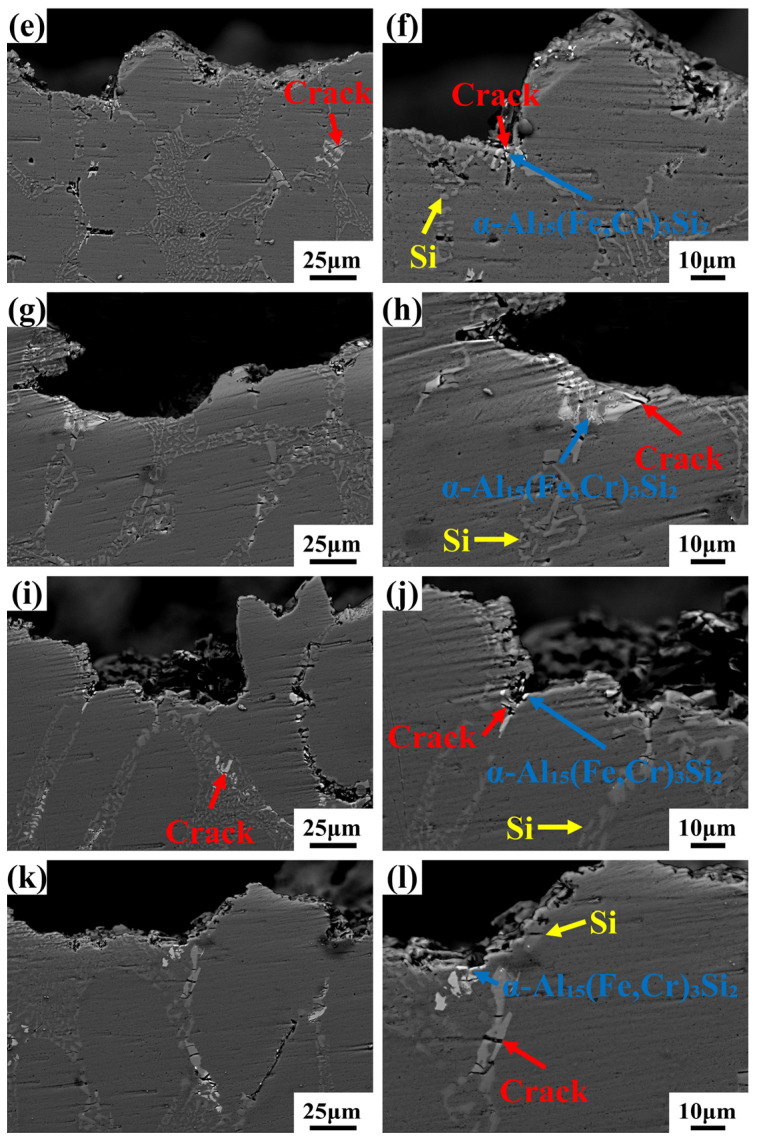
Micrographs of fracture cross-sections: Al-7Si-0.35Mg-0.35Fe with varying Cr: (**a**,**b**) 0Cr; (**c**,**d**) 0.05Cr; (**e**,**f**) 0.10Cr; (**g**,**h**) 0.15Cr; (**i**,**j**) 0.20Cr; (**k**,**l**) 0.25Cr.

To further investigate the nucleation and propagation of deformation cracks under the modification of Fe-rich phases, the morphology of the longitudinal sections of fractured tensile specimens from Al-7Si-0.35Mg-0.35Fe alloys with different Cr contents was examined, as shown in Figure 10. In the 0Cr alloy, cracks primarily nucleated at the interfaces between the needle-like Fe-rich phases and the aluminum matrix and propagated along these interfaces. Some cracks were relatively straight and extended over considerable lengths. This indicates that the bonding between the needle-like β-Al_5_FeSi phases and the α-Al matrix was weakened, making these interfaces prone to crack initiation under external load and providing easy paths for crack propagation. In contrast, for the Cr-containing alloys, cracks were observed at both the Fe-rich phases/Al-matrix and Fe-rich phases/eutectic Si interfaces on the fracture sections. Furthermore, the crack sizes were significantly reduced, particularly in alloys with Cr contents of 0.10–0.20 wt.%. After the addition of the Cr element, the crack propagation no longer extended in a straight line along the phase interface but showed a clearly short and winding expansion path. It was indicated that after the morphology of the Fe-rich phases transformed into a more blunt and smaller-sized α-Al_15_(Fe,Cr)_3_Si_2_, the interfacial bonding strength between it and the aluminum matrix increased, which can better address the adverse effects brought by the needle-like β-Al_5_FeSi. Especially in the 0.20 wt.% Cr alloy, the cracks at the interface were obviously blocked by the massive α phase, and the crack path was more tortuous. However, when the Cr content increased to 0.25 wt.%, excessive Cr led to the coarsening of the α-Al_15_(Fe,Cr)_3_Si_2_ phases. While these coarsened phases contributed to increased strength, they also became potential new sites for crack nucleation, adversely affecting the alloy’s ductility. Therefore, an appropriate addition of Cr transformed the segregated, needle-like β-Al_5_FeSi phases into discrete, more equiaxed α-Al_15_(Fe,Cr)_3_Si_2_ phases, effectively reducing their stress concentration effect. In contrast, excessive Cr addition caused coarsening of the α-Al_15_(Fe,Cr)_3_Si_2_ phases, which further increased strength at the expense of ductility, ultimately leading to a deterioration in the strength-ductility balance.

## 4. Conclusions

Given the low tolerance of Fe content (<0.15 wt.%) in hypoeutectic Al-7Si alloys, this study employed Cr addition to modify the Fe-rich phases in Al-7Si-0.35Mg-0.35Fe alloy with high Fe content, achieving a relatively wide effective addition window. The evolution of Fe-rich phases, as well as the influence of Cr additions with the range of 0–0.25 wt.% on both the microstructural development and the mechanical behavior of the Al-7Si-0.35Mg-0.35Fe alloy, have been investigated. Based on the experimental results, the principal findings are summarized as follows:

(1) Cr addition effectively refines the microstructure and promotes morphological blunting of the Fe-rich phases. With increasing Cr content, the secondary dendrite arm spacing of the alloy initially contracts but subsequently expands beyond a certain threshold, while the Fe-rich phases gradually transform from needle-like β-Al_5_FeSi to short rod-like and blocky α-Al_15_(Fe,Cr)_3_Si_2_ with dispersed distribution. Excessive addition of 0.25 wt.% Cr leads to the coarsening and segregation of the Fe-rich phase.
(2) All Cr-containing alloys exhibited superior mechanical properties, and a broad effective Cr addition range was obtained from 0.05 to 0.20 wt% in the alloy. Compared with the 0Cr alloy, the 0.20Cr alloy achieves approximately a 19% increase in ultimate tensile strength and a 107% increase in elongation, and even. Excessive addition of Cr in 0.25Cr alloy still exhibits increases of approximately 29% in ultimate tensile strength and approximately 35% in elongation.
(3) The fracture surface of the Cr-free alloy primarily consisted of large cleavage planes and coarse microcracks nucleated at the β-Al_5_FeSi/α-Al interface. The fracture surfaces of Cr-containing alloys comprise small cleavage planes, many dimples, and microcracks nucleated at the α-Al_15_(Fe,Cr)_3_Si_2_/α-Al or α-Al_15_(Fe,Cr)_3_Si_2_/eutectic Si interfaces. The blunting morphology of α-Al_15_(Fe,Cr)_3_Si_2_ leads to a shorter and more tortuous path, but the coarsening of the α-Al_15_(Fe,Cr)_3_Si_2_ phase will accelerate the propagation of cracks.

## Figures and Tables

**Table 1:** Chemical composition of alloys with different Cr contents (wt.%).

Alloy	Si	Mg	Fe	Cr	Ti	Sr	Al
0Cr	6.93	0.36	0.33	-	0.10	0.014	Balance
0.05Cr	6.93	0.36	0.31	0.04	0.09	0.010	Balance
0.10Cr	6.93	0.36	0.30	0.08	0.11	0.010	Balance
0.15Cr	6.93	0.38	0.31	0.12	0.10	0.013	Balance
0.20Cr	6.93	0.38	0.31	0.18	0.11	0.010	Balance
0.25Cr	6.83	0.33	0.33	0.23	0.11	0.017	Balance

**Table 2:** EDS analysis of the Fe-rich phase with typical morphologies (at.%).

Point	Morphology	Al	Si	Fe	Cr
1	Needle-like	61.29	22.83	15.88	-
2	Short rod-like	87.69	5.60	4.98	1.73
3	Blocky	63.81	20.42	12.25	3.52
4	Blocky	75.92	7.91	11.55	4.62

## Data Availability

The original contributions presented in this study are included in the article. Further inquiries can be directed to the corresponding authors.
